# Whole process navigated percutaneous endoscopic lumbar discectomy guided by a novel X-ray instant navigation: a technical report and preliminary clinical outcomes

**DOI:** 10.1186/s12891-025-09084-y

**Published:** 2025-10-21

**Authors:** Wenjie Zheng, Wen Xia, Chao Liu, Zhengyang Wu, Minghan Liu, Rui Zuo, Linfeng Mo, Yue Zhou, Changqing Li, Chao Zhang

**Affiliations:** Xinqiao Hospital Orthopedics Department, Army Medical University, No.183, Xinqiao Main Street, Shapingba District, Chongqing, 400037 China

**Keywords:** Percutaneous endoscopic lumbar discectomy, X-ray instant navigation, Permanent calibration, Lumbar disc herniation

## Abstract

**Objective:**

In this study, we established a rapid whole-process Percutaneous Endoscopic Lumbar Discectomy(PELD) surgical workflow guide by a novel X-ray instant Navigation(XIN)based on permanent calibration and specialized instruments. The safety and efficacy of this technique were evaluated through clinical application on surgical treatment in patients with lumbar disc herniation.

**Methods:**

From June 2023 to February 2024, 44 patients with lumbar disc herniation underwent PELD guided by XIN system. A retrospective review of clinical data was conducted, including navigation preparation time, cannulation time, total operative time, intraoperative fluoroscopy exposure, and hospital stay duration. Outcomes were assessed using the Visual Analog Scale (VAS) for back and leg pain, the Oswestry Disability Index (ODI) for functional status, and the modified MacNab criteria for overall efficacy.

**Results:**

The average surgical time was 50.29 min (range: 32–71 min), with an average navigation preparation time of 2.57 min and cannulation placement time of 12.70 min. The average postoperative hospital stay was 1.39 days (range: 1–3 days). The average intraoperative fluoroscopy count was 3.41 times. At the final follow-up, significant improvements were observed in both the VAS scores for back and leg pain and ODI functional impairment index compared to preoperative values, with statistically significant differences (*P* < 0.001). According to the Modified MacNab criteria,27 cases (61.4%) were rated as excellent, 13 cases (29.5%) as good, 3 cases (6.8%) as fair, and 1 patients (2.3%) as poor, resulting in an overall excellent/good rate of 90.9%. No cases of nerve root injury or deep infection occurred.

**Conclusion:**

We developed a whole process navigated PELD technique, incorporating specialized instruments and a streamlined procedural workflow based on X-ray instant navigation (XIN). Preliminary clinical results demonstrate that this technique is safe, efficient, and effective.

## Introduction

Percutaneous endoscopic lumbar discectomy (PELD) is a minimally invasive procedure for intervertebral disc removal, performed through the safe triangular zone of the Cambin triangle [1, 2]. This technique has emerged as one of the mainstream methods for microdiscectomy. Numerous studies have shown [3] that the clinical outcomes of PELD are comparable to those associated with traditional microscopic discectomy techniques. Compared to open discectomy, PELD offers several significant advantages [4, 5], including reduced muscle injury, faster postoperative recovery, shorter hospital stays, and lower infection rates. Consequently, the application of PELD has seen substantial growth in recent years.

PELD requires a unique needle access route, involving ‘blind’ steps for needle placement, dilation, foraminoplasty, and working channels establishment, all guided by fluoroscopy or other imaging techniques [6]. The narrow lumbar foraminal anatomy, combined with the close proximity of critical neurovascular structures, poses significant challenges and increases surgical risks, particularly for less experienced surgeons. Studies show [7, 8] that conventional fluoroscopic guidance in PELD can lead to significant radiation exposure for both patients and the surgical team. In fluoroscopy-guided PELD, the ability to align two-dimensional fluoroscopic images with the underlying three-dimensional anatomy is crucial, making the procedure particularly difficult to master. This complexity often contributes to high early complication rate and prolonged learning curve [9, 10].

Intraoperative navigation technology for spinal surgery provides real-time landmark identification, which can facilitate PELD procedures. However, implementation of various intraoperative spinal navigation may necessitates expensive specialized equipment, technical training, and qualified personnel, along with a lengthy preparation time [11–14].

In our preliminary work, we developed a novel instant spinal navigation system based on permanent calibration of fluoroscopic equipment [15]. By permanently calibrating X-ray fluoroscopy machines, such as C-arms and G-arms, we employed relative algorithms to achieve accurate three-dimensional coordinate systems within the fluoroscopic framework, allowing for instant-use(shoot then navigation mode)of spinal navigation based on two-dimensional x-ray images. This allows for extremely rapid setup of the navigation system, providing results similar to real-time fluoroscopic imaging. Meanwhile, most spinal surgeons are accustomed to performing PELD under two-dimensional fluoroscopic guidance, suggesting a favorable acceptance and adaptability to two-dimensional imaging guidance.

In this study, we are the first to clinically apply permanent calibration-based X-ray navigation technology in spinal surgery and to establish a comprehensive whole-process navigation technique for PELD. This technique employs an X-ray instant navigation system and incorporates a fully navigated set of instruments, including a puncture rod, facet-holding outer sheath, trephine, working channel, and endoscope to assist throughout the surgical procedure. We analyze the efficacy, safety, and clinical value of this technique through a cohort of patients undergoing surgery for lumbar disc herniation.

## Methods

This was a retrospective cohort study conducted at one tertiary hospital in Chongqing, China (Xinqiao Hospital, Army Medical University). Eligible patients were screened, with symptomatic lumbar disc herniation and underwent PELD guided by X-ray instant Navigation based on permanent calibration technology. All surgeries were performed and supervised mainly by five experienced surgeons who were spine dedicated with 10 to 15 years of experience in degenerative lumbar spine surgery. Outcomes were assessed by the Visual Analog Scale (VAS) for back and leg pain, the Oswestry Disability Index (ODI) for functional status [16–18]. The Visual Analog Scale (VAS) score, Oswestry Disability Index (ODI) score, surgical parameters, and all surgery-related adverse events were recorded at baseline (preoperative), at 2 weeks postoperative (first assessment), at 3 months postoperative (second assessment), and at 6 months postoperative (third assessment). Clinical efficacy was assessed using the MacNab criteria (excellent, good, fair, and poor) at the final follow-up. This study was approved by the Research Ethics Committee of Xinqiao Hospital.

### Patients

Between June 2023 and February 2024, a total of 44 patients with symptomatic lumbar disc herniation underwent percutaneous endoscopic lumbar discectomy (PELD) guided by X-ray instant Navigation were enrolled. The detailed inclusion criteria were as follows: (1) age ≥ 18 years, (2) definite diagnosis of typical symptomatic single-level lumbar disc herniation with neurologic signs, including sensory changes, motor weakness, or the presence of abnormal reflex, that corresponded with the preoperative images, including radiographic screening and MRI, (3) no response to appropriate conservative treatment over 3 months. The exclusion criteria were as follows: (1) intraoperative response was not possible because of mental condition, (2) preoperative concomitant spondylolisthesis or deformity warranting correction or fusion, previous lumbar spine surgery, preoperative spinal infection, spinal tumor, or uncontrolled systemic diseases, (3) Patients with less than 6 months follow-up and incomplete clinical data.

## Surgical tools

G-arm(Simm, Inc., Jiangsu, China) Permanent calibration was completed according to the methods described in the literature [15]. Zenta navigation system (Bosscom, Inc., Chongqing, China) Navigation version of transforaminal endoscope system (Bosscom, Inc., Chongqing, China).

## Surgical procedures

### X-ray instant navigation(XIN) assisted PELD

**Fig. 1:**
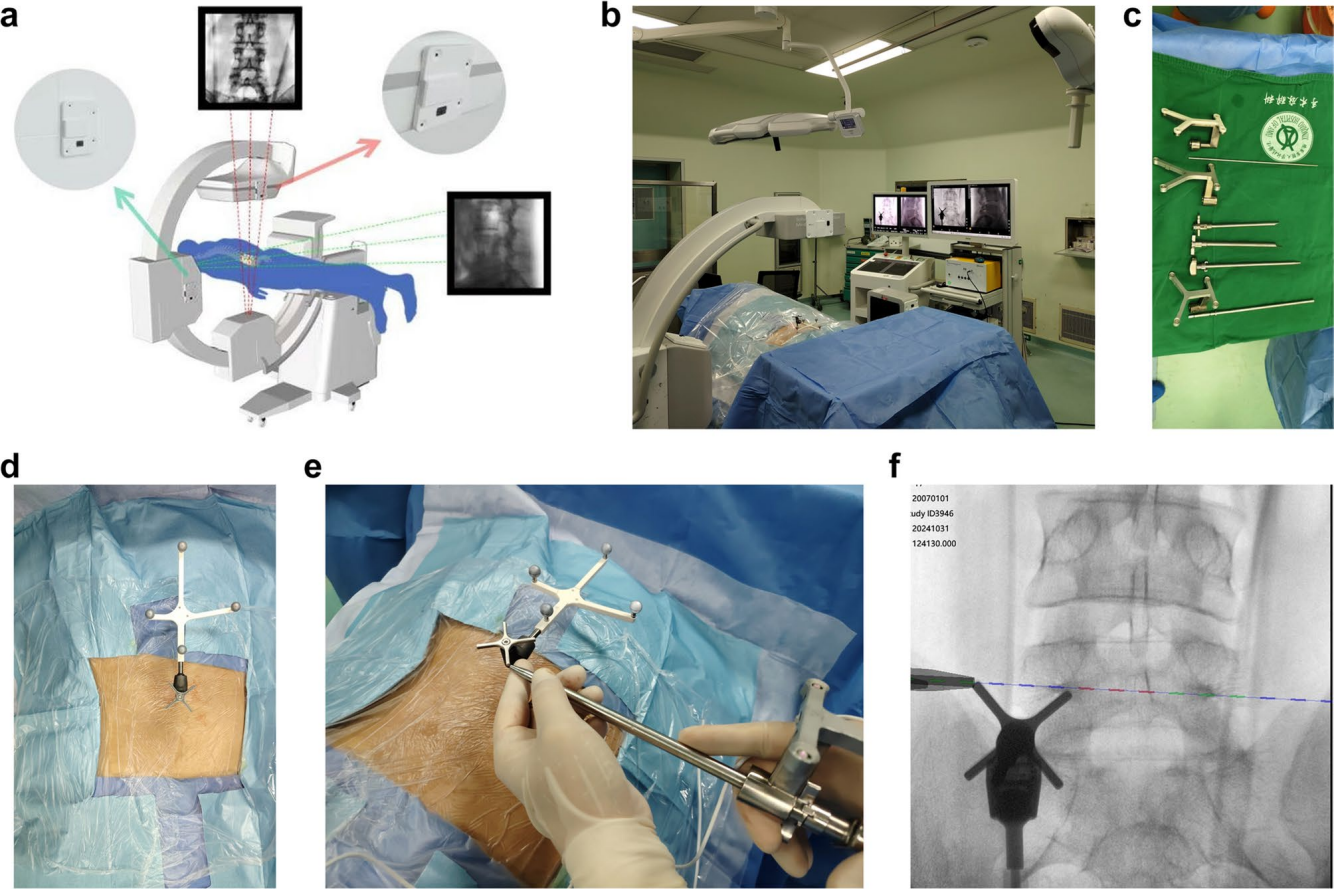
(**a**)The Schematic diagram of the principle of permanent calibration. (**b**)Setup of operative room (**c**)specialized PELD navigation instruments, from left to right: Injectable puncture needle and its tracker, Navigation tracker for outer sheath and working channel, outer sheath, dilator, navigation trephine. (**d**) Installation of the navigation reference frame on the posterior superior iliac spine. (**e**)(**f**) Navigation validation

The patient was placed in the prone position for the procedure. The surgery was assisted by the offline calibrated G-arm and Zenta permanent calibrated 2D navigation system (Fig. 1a, b). The procedure is performed under Monitored Anesthesia Care (MAC) with local anesthesia using 1% lidocaine and sedation, using specialized surgical instruments (Fig. 1c). A small incision was made to secure the cross-shaped needle frame at the posterior superior iliac spine (Fig. 1d). Standard anteroposterior and lateral images were obtained using G-arm fluoroscopy, with the standard lateral view based on the caudal vertebral body if there is any deformity such as scoliosis. The fluoroscopic images were transmitted to the navigation workstation to initiate the navigation process. The navigation preparation typically takes about 2 to 3 min. Intraoperatively, navigation verification can be performed by referring to the specific landmarks of the reference frame (Fig. 1e, f).

**Fig. 2:**
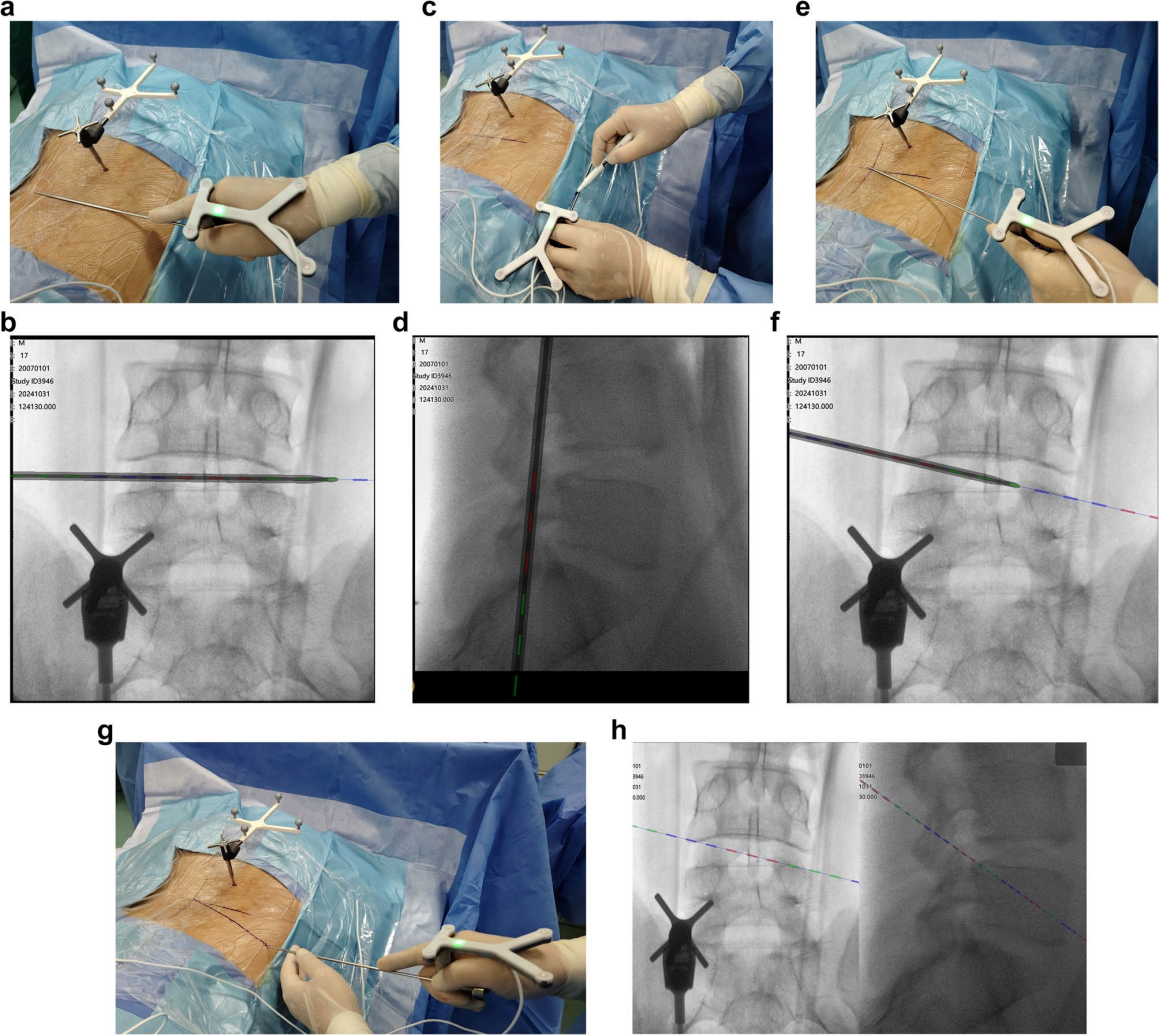
(**a**)(**b**) Localizing the disc space level on the navigation front view (**c**)(**d**) localizing the foramen on the navigation lateral view (**e**)(**f**) Determining the cranial tilt puncture angle. (**g**)(**h**) Designing the puncture trajectory

As shown in Fig. 2. A navigation-guided puncture needle was used to complete the preoperative anatomical calibration, including the intervertebral space (Fig. 2 a, b), the horizontal line of the foramen in the lateral view (Fig. 2 c, d), and the cranial tilt angle of the puncture in AP view (Fig. 2 e, f). The entry point and trajectory were planned on the navigation images referenced the TESSYS technique [19], passing through the tip of superior articular process into the spinal canal (Fig. 2, g, h).

**Fig. 3:**
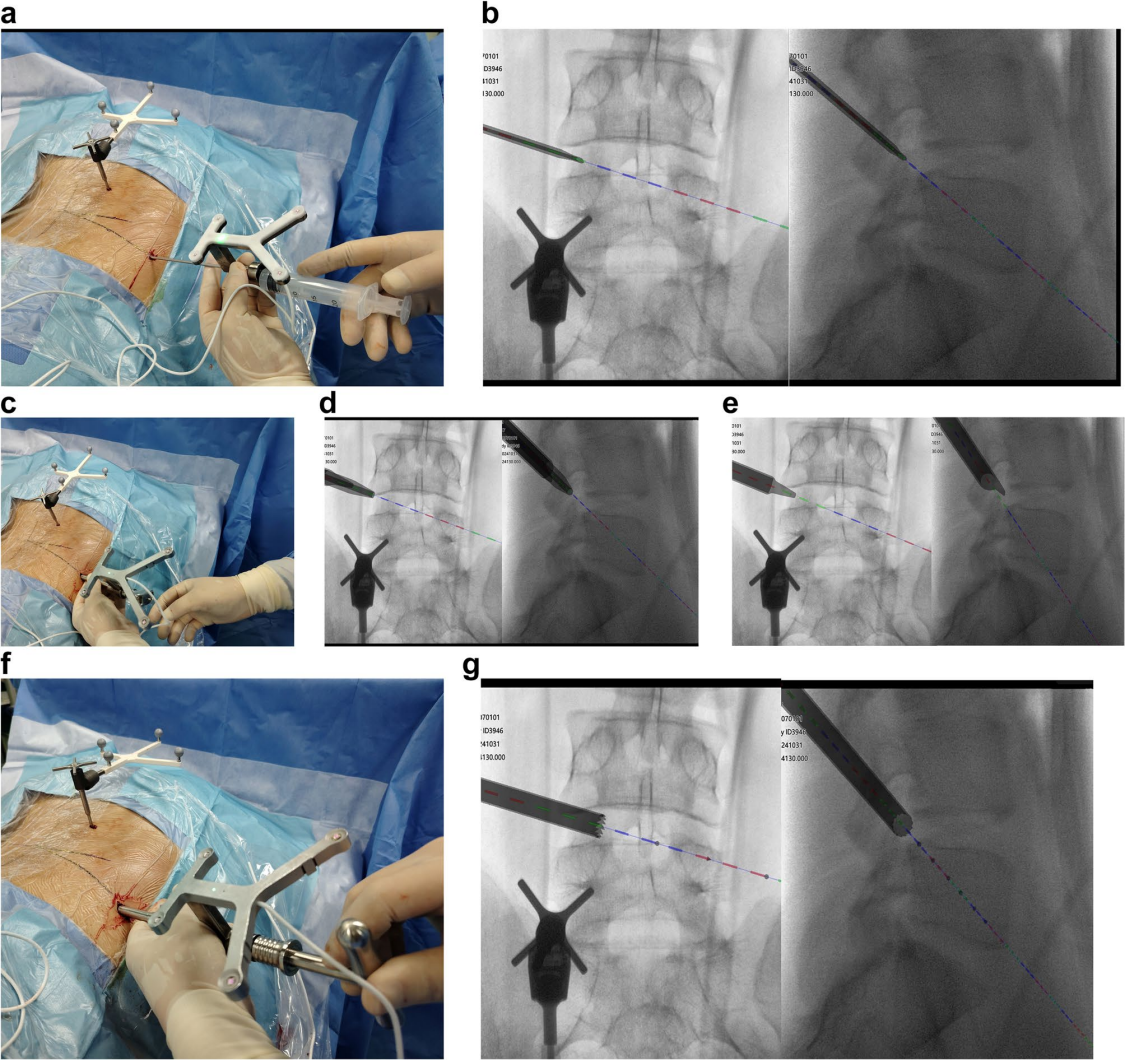
(**a**)(**b**) Navigation-guided transforaminal puncture (**c**)(**d**)(**e**) Navigation-guided dilation and insertion of outer sheath (**f**)(**g**) Foraminoplasty using navigated trephine

A 0.8 cm skin incision was made in entry point. Under navigation guidance, the needle was inserted into the intervertebral foramen along the anterior edge of the superior articular process, with local anesthesia administered along the puncture path (Fig. 3 a, b). The dilation rod and outer sheath was then advanced to the tip of the superior articular process along the needle. The slim frontal ‘tongue’ of the sheath was positioned anterior to the superior articular process along the guide rod to grip the SAP and provide stability for subsequent procedures, while isolating the traversing nerve root(Fig. 3 c, d, e). The navigated trephine was used to complete the foraminoplasty of the superior articular process(Fig. 3 f, g). With the assistance of navigation imaging and the stabilization provided by the outer sheath, the trephine can easily follow the desired trajectory to perform targeted foraminoplasty.

**Fig. 4:**
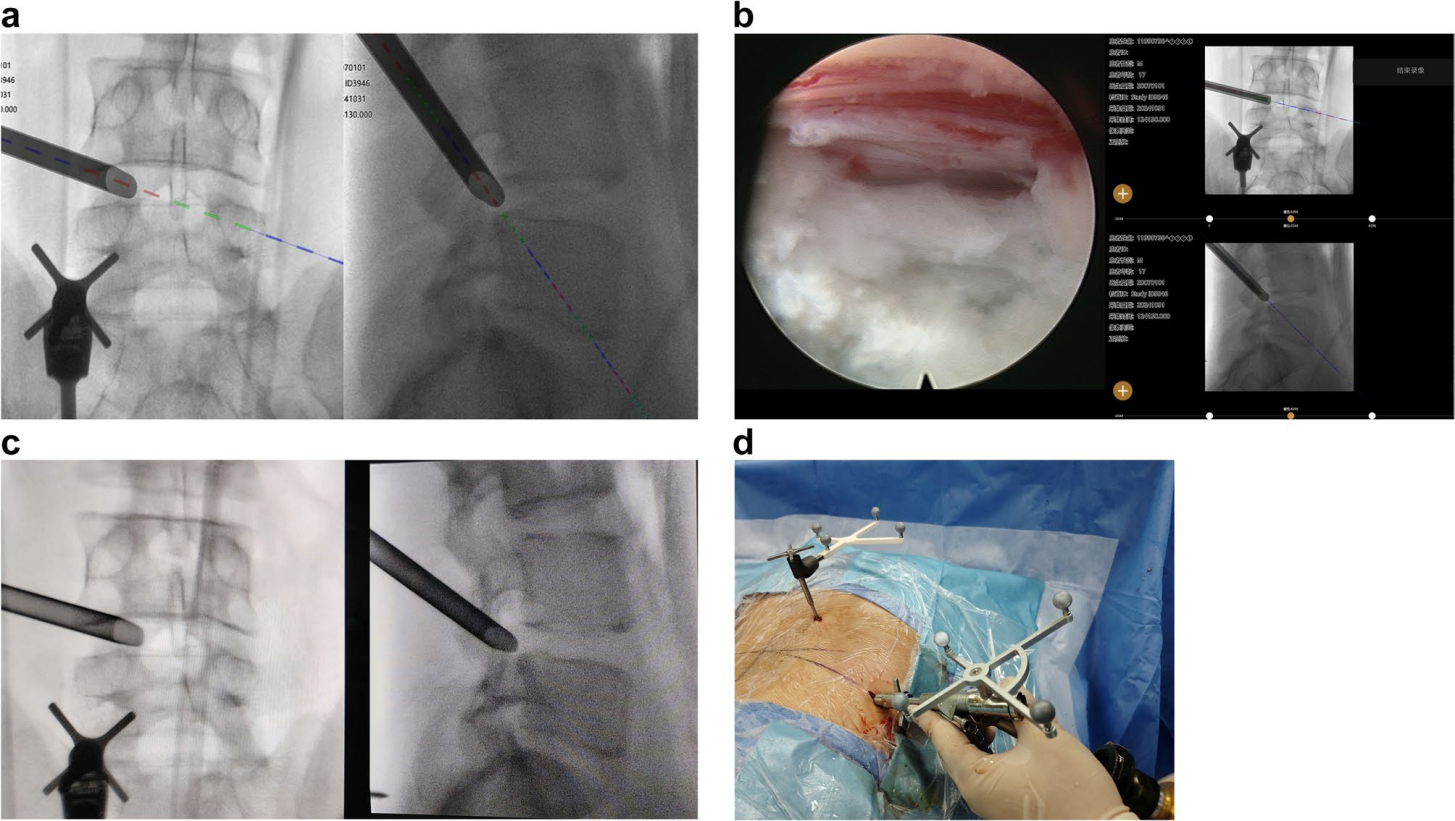
(**a**) navigation view of working channel placement (**b**) fluoroscopic view of working channel placement (**c**)(**d**) prolapsed disc removal using navigated endoscopy

The working channel was inserted also under navigation guidance following foraminoplasty(Fig. 4 a). A fluoroscopy was carried out to verify the position of working cannula(Fig. 4 b), which was then compared with the navigated images. Subsequent procedures, including disc removal and neural decompression, were performed under continuous visual control using a navigated endoscope(Fig. 4 c, d). After discectomy, the working cannula was withdrawn, and the incisions were closed.

## Outcome evaluation

**Fig. 5:**
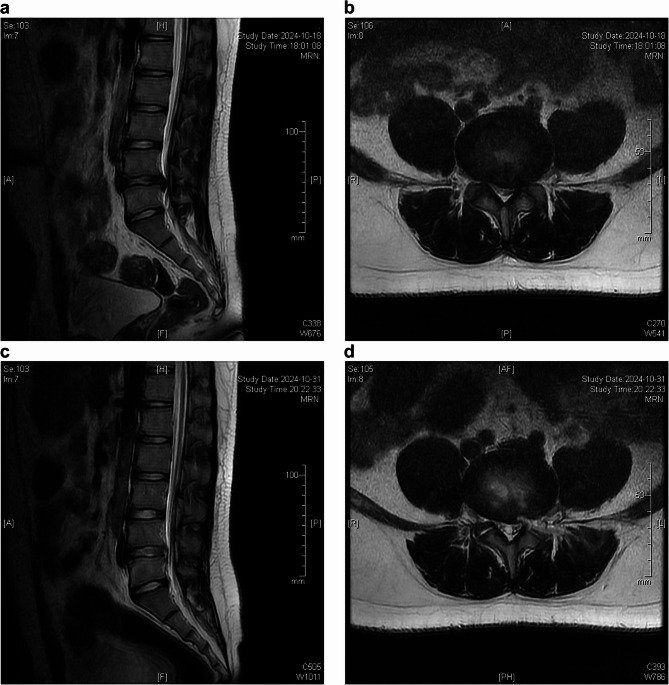
A male patient with lumbar disc herniation at L4,5 underwent navigated PELD surgery (**a**)(**b**) preoperative images of MRI scan (**c**)(**d**) postoperative images of MRI scan

Postoperatively, we performed lumbar CT and MRI scans, which, when compared to preoperative imaging, indicated a successful decompression(Fig. 5). Patient demographics and surgical parameters were recorded. We also documented the navigation preparation time, cannula introduction time and the number of fluoroscopic shots. Navigation preparation time referred to the interval from the installation of the navigation reference frame to the completion of navigation verification, enabling navigational implementation. Cannula introduction time was from the beginning of the incision design in the navigation surgery video to the completion of the navigated working channel placement. The operation time was defined as the duration from making a skin incision (around 8 mm) for the trajectory to wound dressing. We collected visual analog scale (VAS) scores for back and leg pain ranging from 0 to 100 with higher scores indicating more pain, along with Oswestry Disability Index (ODI) scores. Surgical success was assessed using the modified Macnab criteria. Patients’ clinical data were collected by chart reviews and patient-based outcome questionnaires or telephone interviews.

### Statistical analysis

Continuous variables were expressed as means ± standard deviation, preoperative, and postoperative clinical outcomes were compared using paired t-test. P value < 0.05 was considered significant. The IBM SPSS Statistics Ver. 26.0 (IBM Co, Armonk, NY, USA) was used for statistical analysis.

## Results

### Demographic characteristics and surgical data

Between Jun, 2023, and Feb, 2024, 27 men and 17 women (44 patients) were enrolled, with a mean age of 44.0 years (range, 18–86 years). The operated-on levels were L3-4 in 3 patients, L4-5 in 21, and L5-S1 in 20. The mean operative time was 50.29 min (SD = 12.44 min; range, 32–71 min). The mean navigation preparation time was 2.57 min (SD = 1.13 min; range, 1–4 min). The mean cannula placement time was 12.70 min (SD = 5.10 min; range, 4–21 min). The mean hospital stay after the procedure was 1.39 days (SD = 0.58 days; range, 1–3 days). (Tables 1 and 2)

**Table 1:** Demographic characteristics

Characteristics of participants	
Age	
Median(IQR)	44.0(34.5–57.0)
Range	18.0–86.0
Sex	
Male	27
Female	17
Median (IQR) body mass index	24.7(22.4–26.9)
Level	
L3-L4	3
L4-L5	21
L5-S1	20

Data are expressed as median (interquartile range, IQR), counts are used where appropriate

**Table 2:** Surgical data

Perioperative clinical results	
Operation time (mins)	50.29 ± 12.44
Cannula placement time (mins)	12.70 ± 5.10
Navigation preparation time(mins)	2.57 ± 1.13
Postoperative hospital stay (days)	1.39 ± 0.58
Intraoperative fluoroscopy (times)	3.41 ± 1.72

Values are presented as mean ± standard deviation

### Clinical outcomes

The mean preoperative VAS score for leg pain was 66.41 (SD = 16.81). This score improved to 18.82, 15.68, and, finally, 12.39 (SD = 4.17) at postoperative 2 weeks, 3 months, and 6 months, respectively (*P* < 0.001). The mean preoperative VAS score for back pain was 52.98 (SD = 14.96). This score changed to 22.59, 19.38, and, finally, 18.61 (SD = 5.66) at postoperative 2 weeks, 3 months, and 6 months, respectively (*P* < 0.001). The mean preoperative ODI score was 58.77% (SD = 10.36%). The mean postoperative ODI score was 19.09%, 16.89%, and, finally, 15.84% (SD = 4.30%) at postoperative 2 weeks, 3 months, and 6 months, respectively (*P* < 0.001). At the final follow-up review, the MacNab criteria were rated as follows: excellent in 27 patients (61.4%), good in 13 patients (29.5%), fair in 3 patients (6.8%), and poor in 1 patients (2.3%). Therefore, excellent or good results were obtained in 90.9% of the patients, and the rate of symptomatic improvement was 97.7% (Table 3).

**Table 3:** Clinical outcomes

Clinical outcomes	
VAS for leg pain	
Preoperative	66.41 ± 16.81
2 weeks	18.82 ± 6.73 *P* < 0.001
3 months	15.68 ± 5.78 *P* < 0.001
6 months	12.39 ± 4.17 *P* < 0.001
VAS for low back pain	
Preoperative	52.98 ± 14.96
2 weeks	22.59 ± 4.41 *P* < 0.001
3 months	19.38 ± 5.01 *P* < 0.001
6 months	18.61 ± 5.66 *P* < 0.001
Oswestry Disability Index	
Preoperative	58.77 ± 10.36
2 weeks	19.09 ± 4.61 *P* < 0.001
3 months	16.89 ± 4.02 *P* < 0.001
6 months	15.84 ± 4.30 *P* < 0.001
MacNab criteria(at 6 months)	
Excellent	27 61.4%
Good	13 29.5%
Fair	3 6.8%
Poor	1 2.3%

Data are shown as mean ± SD or n (%) unless otherwise indicated

### Operation complications

No major complications including nerve root injury, spinal instability, cerebral spinal fluid leakage, vascular injury, surgical infection was observed in the case series. No revision surgeries were required during the 6-month follow-up period due to symptomatic residual disc herniations or other reasons. Only 1 patient experienced a slight dysesthesia after surgery at the L4/5 level. The symptoms only lasted for 3 days. 1 patient experienced a recurrence of disc herniation at L4/5 six months after surgery and subsequently underwent a repeat MIS-TILF procedure, with symptom relief postoperatively.

## Discussion

The PELD technique has been utilized as a minimally invasive treatment for degenerative lumbar diseases for several decades [3, 20]. The PELD technology, exemplified by the TESSYS technique, achieves effective intra-spinal exploration through foraminoplasty, facilitating the removal of free nucleus pulposus material and direct inspection of neurological structures. Consequently, The TESSYS technique and its modified versions, characterized by the creation of a pathway through foraminoplasty, has become the predominant PELD technique [21, 22].

The TESSYS technique is performed using a posterolateral approach, accomplished through a series of complex maneuvers. It utilizes the Cambin’s safe triangle to achieve transforaminal puncture, followed by soft tissue dilation and foraminoplasty. Effective foraminoplasty helps establish an optimal pathway for the targeted resection of the intervertebral disc and facilitates intra-spinal exploration. However, the foraminal structures of the lumbar spine are narrow, and important neurovascular elements are closely adjacent, necessitating a high level of precision during the procedure. This anatomical constraint also presents significant potential risks [9].

The establishment of the surgical pathway in PELD procedures is performed under non-visual conditions and requires imaging guidance. “Blind” procedures necessitate a profound understanding of the anatomical approach and relevant imaging anatomy, as well as skilled surgical techniques and extensive experience [23].

Fluoroscopy-guided surgery poses significant challenges, including prolonged operative time, a prolonged learning curve, and considerable radiation exposure, which have been substantial barriers to the early adoption of PELD [24, 25]. Therefore, in addition to fluoroscopic guidance, many researchers have explored alternative, more efficient imaging modalities to assist in performing the procedure.

In our study, the average surgical time was 50.29 min (range: 32–71 min), with an average navigation preparation time of 2.57 min and cannulation placement time of 12.70 min. Our navigation system can achieve instant image registration during fluoroscopy, with the entire process completed within a matter of seconds. This is a remarkable improvement over the past, where registration times typically spanned from a few minutes to as long as 10 min. The operation of this system is as straightforward as conventional fluoroscopy, with one-click data transfer, necessitating minimal training for operators and reducing the need for specialized personnel assistance.

The average postoperative hospital stay was 1.39 days (range: 1–3 days), and the average intraoperative fluoroscopy count was 3.41 times. At the final follow-up, significant improvements were observed in both the VAS scores for back and leg pain and ODI functional impairment index compared to preoperative values, with statistically significant differences (*P* < 0.001). According to the Modified MacNab criteria, 27 cases (61.4%) were rated as excellent, 13 cases (29.5%) as good, 3 cases (6.8%) as fair, and 1 patient (2.3%) as poor, resulting in an overall excellent/good rate of 90.9%. Notably, no cases of nerve root injury or deep infection occurred. Further studies with larger sample sizes and longer follow-up periods may help to validate these findings and provide more comprehensive insights into the clinical application.

Recently, intraoperative spinal navigation technology offers real-time landmark identification, greatly enhancing the convenience of spinal minimally invasive surgeries. Numerous studies have confirmed that navigation enhances accuracy in spinal surgery, reduces radiation exposure, and improves surgical efficiency and outcomes [12, 26, 27].

Many researchers have explored various imaging techniques, including intraoperative computed tomography (CT) [27], electro-magnetic navigation [9], 3D intraoperative navigation [10], MRI [28], and ultrasound [29], or even combined technique [30] to assist in the blind steps of PELD, such as needle insertion, dilation, and foraminoplasty. Related literature consistently supports that navigational guidance can streamline operations’ accuracy and speed in the blind section of PELD, thereby accelerating the surgical process and even optimizing the learning curve [31].

Despite the potential benefits of navigation systems, navigated endoscopic techniques remain relatively niche and have not yet gained widespread adoption. We summarize that several factors contribute to this situation from literatures [26]: the high cost of intraoperative navigation equipment, the complexity of operational techniques, elevated technological demands requiring further specialized training and support, a lack of dedicated navigational surgical instruments, and prolonged preparation times.

The complex navigation preparation steps and the additional time required for spinal navigation preparation may be one of the key reasons why it is difficult to widely adopt a navigated PELD procedure. Numerous studies suggest [32, 33] that navigation can expedite certain aspects of surgical procedures; however, the calibration and preparation steps involved in navigation are often complex and time-consuming, potentially prolonging the overall duration of surgery. A critical factor contributing to this issue is the inconsistency of X-ray machine parameters, as variations in gravity and positioning can lead to unpredictable alterations in the direction of the X-ray source and its distance from the imaging intensifier [34]. Consequently, both intraoperative 2D fluoroscopic navigation and preoperative 3D CT-based navigation methods struggle to adapt to the stochastic changes in the spatial geometric parameters of the X-ray machine [34]. As a result, multiple intraoperative recalibrations are often required prior to navigation implementation to prevent navigational failure, frequently necessitating the use of cumbersome auxiliary devices [35]. These factors inevitably result in a cumbersome calibration process, consume valuable operating space, and ultimately lead to reduced surgical efficiency.

To address the issues of cumbersome and time-consuming navigation preparation processes, we have developed [15] a novel permanent calibration technique for spinal surgery navigation. By installing self-developed optical trackers on the fluoroscopy machine (C/G/O arm), this method facilitates rapid and precise intraoperative spatial positioning of the X-ray source and corresponding perspective images in any spatial orientation. Once a specific X-ray machine is permanently calibrated, automated image and instrument registration can be accomplished instantly after fluoroscopic image capture and transfer. Therefore, we have named it the Permanently Calibrated (PerCal) Instant Navigation Technique. By utilizing this navigation technique, the preparation process is extremely simple and quick; navigation can begin after a few straightforward steps, including installing the reference frame, conducting conventional fluoroscopy, and transmitting the images. In this study, the navigation preparation time we measured was less than three minutes. To our knowledge, this is the fastest and most straightforward spinal navigation system in the world.

A review of extensive literature on navigated surgery highlights the essential role of specialized instruments in ensuring successful completion of key steps. While many researchers are investigating specific whole-process navigational approaches, true whole-process navigation involves more than just whole-process guidance [36] The use of dedicated navigation instruments for critical stages can streamline the process, expedite the surgical workflow, and improve accuracy. The establishment of the cannulation pathway in PELD requires the sequential use and coordination of various surgical instruments, including puncture needles, rods, dilating tubes, trephine, and working channels. In previous navigation-guided and robot-assisted PELD surgeries [36], the range of navigational instruments has been limited, predominantly using a single navigation rod or puncture needle for guidance. Only in the electro-magnetic navigation PELD surgeries reported by Wu [37]. Navigation-guided puncture represents only a small part of PELD procedures, while the critical step of foraminoplasty is essential for establishing the final pathway [38]. Furthermore, monitoring the depth and trajectory of the trephine is particularly important for the effectiveness and safety of the procedure, which makes the navigational visualization of the trephine especially necessary. Additionally, the extension line of any navigated instrument can assist the surgeon in determining the final intro-canal position facilitating the design of an optimal angulation and direction. In most literature [34, 35] on navigation- or robot-assisted PELD surgeries, it is not specified how subsequent procedures, such as foraminopalsty and channel insertion, are performed under navigation after initial navigated puncture/dilation. We speculate that this part of the procedure may have been performed under fluoroscopic guidance instead of dangerous blind manipulation, which somewhat diminishes the value and efficacy of intraoperative navigation.

We designed a navigation facet-holding channel with a long “tongue” that allows for real-time observation, aiming to enhance the stability and safety of the trephine foraminoplasty. Instruments slipping on the sloped surfaces of bone is a common issue and concern in navigation-guided surgeries [39]. The irregular sloped surfaces of the lateral facets of the lumbar vertebrae increase the risk of slipping and drifting, leading to deviations in the approach during foraminoplasty and potentially posing risks to the neural roots. We initially [19] used a specialized dual-channel pipeline and K-wire for auxiliary fixation in navigation PELD, which helps stabilize the trephine. Normally, ensuring the correct positioning of the pipeline and K-wire requires additional manipulation and fluoroscopic confirmation, complicating the surgical process. The “tongue” portion of the external sheath was positioned between the annulus fibrosus and the ventral side of the superior articular process, serving as a fulcrum that enhanced the stability of the trephine during foraminoplasty and prevented slippage and drifting. At the same time, the exiting nerve roots were isolated by external sheath, ensuring their safety. In addition, the channel isolates the richly innervated structures of the posterior longitudinal ligament, which also helps reduce local pain during the foraminoplasty. The insertion of the external channel is also performed under navigation along the puncture rod to the correct position. The most critical and dangerous part of the foraminoplasty is then completed under continuous visibility in navigation monitoring. The direction and depth of the trephine are constantly controllable. After the foraminoplasty, the placement of the navigated working channel and the use of the navigated endoscope assist the surgeon in maintaining awareness of the surgical target at all times.

Overall, we have achieved full-process navigation for all key aspects, including puncture, dilation, holding channel, trephine, working channel, and endoscope. Consequently, there is no longer a need for fluoroscopic observation of the positions of the relevant instruments during the procedure. Under the guidance of true full-process navigation, the accuracy and convenience of the PELD procedure have greatly increased, eliminating the need for additional intraoperative fluoroscopy. Most cases can be completed after a single fluoroscopic registration of the navigation, allowing all operations to be performed efficiently. In addition, the patients in our study who underwent surgery appeared to have fewer fluoroscopy times, and no intraoperative nerve injury occurred.

Limitation: This is a series of technical reports on the full-process navigation-assisted PELD surgery. The number of cases is relatively small and limited to a single center, and no prospective controlled studies were conducted. However, the early results are promising, particularly the very short navigation preparation time and the fully navigated process, which undoubtedly holds great appeal for surgeons and has the potential for broader promotion and application. Additionally, the use of local anesthesia in navigated surgeries remains controversial, and the navigation accuracy under local anesthesia requires specialized follow-up validation through clinical trials.

## Conclusion

In this study, we firstly implemented an instant navigation system in spine surgery to facilitate the performance of PELD. A dedicated surgical workflow was established, and preliminary results confirmed that this technical system is rapid, safe, and efficient

## Data Availability

The datasets used and analysed during the current study are available from the corresponding author on reasonable request.

## References

[1] Ahn Y. Endoscopic spine discectomy: indications and outcomes. Int Orthop. 2019;43(4):909–16. 30612170 10.1007/s00264-018-04283-w

[2] Lei Wang T, Wang, Ning F, et al. Clinical outcome of percutaneous endoscopic lumbar decompression in treatment of elderly patients with lumbar spinal stenosis: a matched retrospective study. Int Orthop. 2024;48(1):201–9. 37632530 10.1007/s00264-023-05947-y

[3] Pan M, Li Q, Li S, Mao H, et al. Percutaneous Endoscopic Lumbar Discectomy: Indications Complications Pain Physician. 2020;23(1):49–56. 32013278

[4] Pravesh SG, Sidney MR, Wilco CP, et al. Full endoscopic versus open discectomy for sciatica: randomised controlled non-inferiority trial. BMJ. 2022;376:e065846. 35190388 10.1136/bmj-2021-065846 PMC8859734

[5] Li H, Xiao C, Pan H, et al. Surgical strategy for lumbar disc herniation based on the MSU classification: percutaneous endoscopic lumbar discectomy versus transforaminal lumbar interbody fusion: a 5-year retrospective study. Orthop Surg. 2024;16(8):1963–73. 38961670 10.1111/os.14145 PMC11293921

[6] Sivakanthan S, Hasan S, Hofstetter C. Full-endoscopic lumbar discectomy. Neurosurg Clin N Am. 2020;31(1):1–7. 31739919 10.1016/j.nec.2019.08.016

[7] Somma F, D’Agostino V, Negro A, et al. Radiation exposure and clinical outcome in patients undergoing percutaneous intradiscal ozone therapy for disc herniation: fluoroscopic versus conventional CT guidance. PLoS One. 2022;17(3): e0264767. 35290390 10.1371/journal.pone.0264767 PMC8923460

[8] Ahn Y, Kim CH, Lee JH, et al. Radiation exposure to the surgeon during percutaneous endoscopic lumbar discectomy: a prospective study. Spine (Phila Pa 1976). 2013;38:617–25. 23026867 10.1097/BRS.0b013e318275ca58

[9] Lee DY, Lee SH. Learning curve for percutaneous endoscopic lumbar discectomy. Neurol Med Chir (Tokyo). 2008;48(9):383–8. 18812679 10.2176/nmc.48.383

[10] Wu B, Wei T, Yao Z, et al. A real-time 3D electromagnetic navigation system for percutaneous transforaminal endoscopic discectomy in patients with lumbar disc herniation: a retrospective study. BMC Musculoskelet Disord. 2022;23(1): 57. 35039040 10.1186/s12891-022-05012-6 PMC8764808

[11] Alluri RK, Sivaganesan A, Vaishnav AS, Dupon M, Qureshi SA. Surface navigation and the influence of navigation on MIS surgery. Global Spine J. 2022;12(Suppl 2):19S–26S. 10.1177/21925682211028587 PMC8998479 35393880

[12] John PW, Lane F, Caleb S, et al. Image-Guided navigation in spine surgery: from historical developments to future perspectives. J Clin Med. 2024;13:7. 10.3390/jcm13072036 PMC11012660 38610801

[13] Karthik T, Justin EB, Elsa MA, et al. Multimodality imaging for 3D printing and surgical rehearsal in complex spine surgery. Radiographics. 2024;44(3):e230116. 38386600 10.1148/rg.230116 PMC10924222

[14] Pei X, Feng F, Jun C, et al. Real-time ultrasonography-magnetic resonance image fusion navigation for percutaneous transforaminal endoscopic discectomy. J Neurosurg Spine. 2020;33(2):192–8. 32217792 10.3171/2020.1.SPINE191223

[15] Wu ZY, Shu YC, Ling J, et al. Percal: a novel permanent calibration method for spinal surgery navigation. IEEE Trans Instrum Meas. 2024;73:1–15.

[16] La Rocca G, Mazzucchi E, Pignotti F, et al. Intraoperative CT-guided navigation versus fluoroscopy for percutaneous pedicle screw placement in 192 patients: a comparative analysis. J Orthop Traumatol. 2022;23(1): 44. 10.1186/s10195-022-00661-8. 36048284 10.1186/s10195-022-00661-8 PMC9437178

[17] Kim JH, Jitpakdee K, Kotheeranurak V, et al. Is navigation beneficial for transforaminal endoscopic lumbar foraminotomy? A preliminary comparison study with fluoroscopic guidance. Eur Spine J. 2023;32(8):2808–18. 10.1007/s00586-023-07624-5. 36920512 10.1007/s00586-023-07624-5

[18] Huang X, Gong J, Liu H, et al. Unilateral biportal endoscopic lumbar interbody fusion assisted by intraoperative O-arm total navigation for lumbar degenerative disease: a retrospective study. Front Surg. 2022;9: 1026952. 10.3389/fsurg.2022.1026952. 36211257 10.3389/fsurg.2022.1026952 PMC9539070

[19] Schubert M, Hoogland T. Endoscopic transforaminal nucleotomy with foraminoplasty for lumbar disk herniation. Oper Orthop Traumatol. 2005;17(6):661. 10.1007/s00064-005-1156-9 16369758

[20] Chang IJ, Seung ML. Complications and management of endoscopic spinal surgery. Neurospine. 2023;20(1):56–77. 37016854 10.14245/ns.2346226.113 PMC10080410

[21] Zhi P, Yoon H, Seong Y, Kai C. Efficacy of transforaminal endoscopic spine system (TESSYS) technique in treating lumbar disc herniation. Med Sci Monit. 2016;22:530–9. 10.12659/MSM.894870 PMC4762298 26887645

[22] Zhang Y, Chu J, Xia Y, et al. Research trends of percutaneous endoscopic lumbar discectomy in the treatment of lumbar disc herniation over the past decade: A bibliometric analysis. J Pain Res. 2023;16:3391–404. 37814606 10.2147/JPR.S421837 PMC10560474

[23] Zhao Y, Bo X, Wang C, et al. Guided punctures with ultrasound volume navigation in percutaneous transforaminal endoscopic discectomy: a technical note. World Neurosurg. 2018;119:77–84. 10.1016/j.wneu.2018.07.185. 10.1016/j.wneu.2018.07.185 30071330

[24] Hsu HT, Chang SJ, Yang SS, Chai CL. Learning curve of full endoscopic lumbar discectomy. Eur Spine J. 2013;22:727–33. 23076645 10.1007/s00586-012-2540-4 PMC3631049

[25] Iprenburg M, Wagner R, Godschalx A, Telfeian AE. Patient radiation exposure during transforaminal lumbar endoscopic spine surgery: a prospective study. Neurosurg Focus. 2016;40(2):E7. 26828888 10.3171/2015.11.FOCUS15485

[26] Heydar AM, Tanaka M, Prabhu SP, Komatsubara T, et al. The impact of navigation in lumbar spine surgery: A study of historical aspects, current techniques and future directions. J Clin Med. 2024;13(16): 4663. 39200805 10.3390/jcm13164663 PMC11354833

[27] Quillo-Olvera J, Lin GX, Suen TK, Jo HJ, Kim JS. Anterior transcorporeal tunnel approach for cervical myelopathy guided by CT-based intraoperative spinal navigation: technical note [Technical note]. J Clin Neurosci. 2018;48:218–23. 29174757 10.1016/j.jocn.2017.11.012

[28] Marker DR, Thainual U, Ungi P. 1.5 T augmented reality navigated interventional MRI: paravertebral sympathetic plexus injections. Diagn Interv Radiol. 2017 May-Jun;23(3):227–32. 28420598 10.5152/dir.2017.16323 PMC5411005

[29] Chi M, Chen AS. Ultrasound for lumbar spinal procedures. Phys Med Rehabil Clin N Am. 2018;29(1):49–60. 29173664 10.1016/j.pmr.2017.08.005

[30] Xie P, Feng F, Cao J, et al. Real-time ultrasonography-magnetic resonance image fusion navigation for percutaneous transforaminal endoscopic discectomy. J Neurosurg Spine. 2020;33(2):192–8. 32217792 10.3171/2020.1.SPINE191223

[31] Hao Q, Li D, Lin X, et al. Puncture and localization for percutaneous endoscopic lumbar discectomy with C-arm navigation: a randomized controlled cadaver trial. Ann Transl Med. 2021;9(23):1730. 35071424 10.21037/atm-21-5844 PMC8743699

[32] Oba H, Ikegami S, Kuraishi S, et al. Perforation rate of pedicle screws using hybrid OR combined with intraoperative CT navigation for adolescent idiopathic scoliosis: impact of distance from the reference frame and other risk factors. Spine (Phila Pa 1976). 2020;45:E1357–64. 32890303 10.1097/BRS.0000000000003673

[33] Jin MR, Lei LY, Li FQ, et al. Does Robot Navigation and Intraoperative Computed Tomography Guidance Help with Percutaneous Endoscopic Lumbar Discectomy? A Match-Paired Study. World Neurosurg 2021 Mar;147:e459-67. 10.1016/j.wneu.2020.12.095 33385595

[34] Qin H, Huang SB, Xu L, et al. Radiation Exposure and Operation Time in Percutaneous Endoscopic Lumbar Discectomy Using Fluoroscopy-Based Navigation System. World Neurosurg 2018 Jul;127:e39-48. 10.1016/j.wneu.2019.01.289 30802551

[35] Adetokunbo AO, Jared F, David BC, et al. Minimally invasive direct lateral, retroperitoneal transforaminal approach for large L1–2 disc herniations with intraoperative CT navigational assistance: technical note and report of 3 cases. J Neurosurg Spine. 2018;29:46–53. 29676674 10.3171/2017.11.SPINE17509

[36] Lee YS, Cho DC, Kim KT. Navigation-guided/robot-assisted spinal surgery: a review article. Neurospine. 2024;21(1):8–17. 38569627 10.14245/ns.2347184.592 PMC10992634

[37] Wu J, Ao S, Liu H, Wang W, Zheng W, Li C, Zhang C, Zhou Y. Novel electromagnetic-based navigation for percutaneous transforaminal endoscopic lumbar decompression in patients with lumbar spinal stenosis reduces radiation exposure and enhances surgical efficiency compared to fluoroscopy: a randomized controlled trial. Ann Transl Med. 2020;8(19):1215. 33178747 10.21037/atm-20-1877 PMC7607128

[38] Soliman H, Fridley J, Telfeian A, et al. Minimally invasive, Far lateral lumbar microdiscectomy with intraoperative computed tomography navigational assistance and electrophysiological monitoring[J]. World Neu Surg. 2019;122:E1228–39. 10.1016/j.wneu.2018.11.020 30447467

[39] Liu JB, Wu JL, Zuo R, Li CQ, Zhang C, Zhou Y. Does MIS-TLIF or TLIF result in better pedicle screw placement accuracy and clinical outcomes with navigation guidance? BMC Musculoskelet Disord. 2022;23(1):153. 10.1186/s12891-022-05106-1. 35172784 10.1186/s12891-022-05106-1 PMC8848978

